# Special Issue “Bioinformatics of Unusual DNA and RNA Structures”

**DOI:** 10.3390/ijms25105226

**Published:** 2024-05-10

**Authors:** Martin Bartas, Václav Brázda, Petr Pečinka

**Affiliations:** 1 Department of Biology and Ecology, Faculty of Science, University of Ostrava, 710 00 Ostrava, Czech Republic; petr.pecinka@osu.cz; 2 Institute of Biophysics, Czech Academy of Sciences, Královopolská 135, 612 00 Brno, Czech Republic; vaclav@ibp.cz

## 1. Introduction

Nucleic acids are not only static carriers of genetic information but also play vital roles in controlling cellular lifecycles through their fascinating structural diversity. Gone are the days when DNA was perceived as a right-handed double helix and mediator RNA was imagined mostly as a linear single-stranded molecule. We now know that various structures, including quadruplexes, triplexes, and looped and zig-zagged structures, dynamically interact with proteins and cooperate in fine-tuning molecular processes. Section 2 of this Editorial is an overview of this exciting field of science and serves as a short guide to the topic of “unusual” DNA and RNA structures. In Section 3, we briefly summarize and highlight the main messages in the eight impactful articles published in this Special Issue, each of them representing various and important aspects of current research efforts. Finally, Section 4 discusses the current and future directions and implications for applications.

## 2. Overview of Unusual Nucleic Acid Structures

Unusual nucleic acid structures (UNas) can be defined as noncanonical nucleic acids differing from the classical double-stranded structure of B-DNA and are mostly abbreviated to non-B DNA (a term introduced in the early 80s [1]). However, this may be quite misleading, as these structures often arise also in RNA or DNA–RNA hybrid molecules (hence our use of the novel term “unusual nucleic acid structures”, abbreviated to UNas). The division of UNas may be quite tricky; historically, the most traditional categories are duplexes [2,3], triplexes [4], and quadruplexes [5]. In addition, all these “-plexes” can be intramolecular (containing only a single nucleic acid strand) or intermolecular (wherein two or more nucleic acid strands are involved) [6,7]. Unusual duplexes are mostly represented by the A-form [8] and Z-form of nucleic acids [9]. Triplexes are characterized by a triple-helical DNA structure in the case of intramolecular DNA triplexes (sometimes called H-DNA) [10] or by noncoding RNA pairing with DNA duplexes through Hoogsteen interactions in the case of intermolecular triplexes [11,12]. Quadruplexes can be divided into G-quadruplexes and i-motifs [13]. Aside from these, there are also somewhat special UNas called R-loops (three-stranded structures consisting of a DNA–RNA hybrid and a displaced strand of DNA) [14] and cruciforms, the latter of which form four-way-junction, double-stranded-stems, and single-stranded loops [15]. All the abovementioned UNas are depicted in Table 1, and key information about them is summarized.

It is worth mentioning that the in vivo existence (and potential biological relevance) of even-higher-order “-plexes” cannot be excluded. Such structures could then be named pentaplexes, sextuplexes, and so on [43].

The formation of most UNas is dependent on primary sequence information [44]; specific UNa-forming sequential motifs have been discovered and experimentally validated (Table 1). Thanks to the rapid development of computer technology and capacities, it is now possible to predict the occurrence of UNa-forming sequences in the whole eukaryotic chromosomes relatively easily, and several user-friendly web servers have been developed for this purpose. One such example allows the prediction of G-quadruplex-forming sequences [45], cruciform-forming sequences [46], R-loop-forming sequences [47], and Z-DNA-forming sequences: the DNA analyzer web server (https://bioinformatics.ibp.cz/#/, accessed on 29 April 2024).

## 3. Current Research Highlights

Here, we would like to shortly highlight the main findings of works published in this Special Issue and encourage readers to read through the articles in their entirety.

Anthony Mittermaier and his team developed a very useful (and user-friendly) web server that allows users to analyze multiplicities of their provided G-quadruplex-forming sequences (https://www.mcgill.ca/mittermaierlab/greg-webserver, accessed on 29 April 2024). In addition, their article presents a detailed bioinformatic survey of the G-quadruplex polymorphism in human gene promoter regions, linking G-quadruplex polymorphisms to biological functions and providing new criteria with which to identify biologically relevant G-quadruplex-forming regions [48].

Another freely accessible service was developed by Jiří Šťastný’s group from Mendel University. Existing computer programs cannot easily predict where R-loops might occur in DNA. To address this, a new tool called R-loop tracker was developed (https://bioinformatics.ibp.cz/#/analyse/rloopr, accessed on 29 April 2024). This free web-based tool can predict R-loops in genomic DNA and allows researchers to compare these predictions to other DNA analyses [47].

It was previously found that G-quadruplexes can arise in long noncoding RNAs (lncRNAs) [33]. The study by Singh et al. identified lncRNA clusters with G4-forming sequences in cervical cancer patients, confirmed the formation of G-quadruplexes in specific lncRNAs, and discussed their roles as potential prognostic biomarkers for cervical cancer [49].

A recent article by Nicoletto et al. discusses the presence, conservation, and localization of putative G-quadruplex-forming sequences in human arboviruses [50]. Arboviruses are transmitted by arthropod vectors (arthropod-borne viruses, i.e., arboviruses) and comprise many important human pathogens, including Dengue virus, West Nile virus, Zika virus, or Tick-borne encephalitis virus [51]. Their study reveals the predominant locations of G-quadruplex-forming sequences in coding sequences and three-prime untranslated regions (3′UTRs). It also highlights their regulatory roles, emphasizing the potential of using G-quadruplex structures as antiviral targets [50].

The article by Gumina and Richardson et al. discusses the role of G-quadruplexes and the DHX36 helicase in gene expression regulation, particularly in cancer cells, highlighting the impact of DHX36 knockout on gene expression associated with G-quadruplex content in promoters or gene regions [52]. The findings suggest that DHX36 knockout leads to subtle but widespread changes in gene expression and provides valuable insights into the complex interplay between G-quadruplex structures, helicases like DHX36, and gene expression regulation, especially in the context of cancer [52].

The study by Feng, Luo et al. explores the effects of potassium (K^+^) and sodium (Na^+^) ions on global G-quadruplex formation in rice (*Oryza sativa*) [53]. The authors utilized a high-throughput method called BG4-DNA-IP-seq (DNA immunoprecipitation with anti-BG4 antibody coupled with sequencing). One of the exciting findings is that K^+^-specific G-quadruplexes are more associated with active histone marks and low DNA methylation levels compared to Na^+^-specific G-quadruplexes. This important research will facilitate the functional characterization of G-quadruplexes in rice and allow the potential use of specific G-quadruplex locations for biotechnological advancements in the future [53].

The study by Shavkunov et al. focused on tRNA fragments (tRFs) and their roles in interspecies interactions in bacterial communities [54]. Their research highlighted the emergence of novel types of RNAs and their potential significance in bacterial and eukaryotic cells [54].

The last published work in this Special Issue is a thorough review by Zulfiqar et al. dealing with Virus-Induced Gene Silencing (VIGS), mainly with respect to crop improvement [55]. VIGS represents a powerful tool for analyzing gene function and inducing heritable epigenetic modifications. This review also highlights the role of VIGS in developing crop varieties with improved agronomic traits and stress tolerance [55].

## 4. Future Perspectives

There is a growing number of bioinformatic tools for UNas prediction and biophysical characterization [45,56,57,58,59] as well as for determining their roles in various diseases including cancer [60,61]. Contemporary, specific antibodies against cruciforms [62,63], left-handed nucleic acids [64], G-quadruplexes [65], and i-motifs [66] have also been developed, allowing effective analyses of UNas both in vitro and in situ. Even if UNas are often difficult to sequence, current methods and their modifications [67] allow accurate sequencing and determination in genomes, leading to the finalization of telomere-to-telomere gapless assemblies [68,69,70,71]. Although there are currently many tools for the prediction and experimental validation of UNas, their structural bioinformatical characterization or modeling is somewhat lagging. In the field of protein science, there are currently many approaches for the de novo (ab initio) prediction of structures only from the amino acid sequences, e.g., AlphaFold [72] or trRosetta [73], and they are also usually accessible via a user-friendly interface [73,74]. In the case of UNas, no such straightforward method exists so far. Although some pioneering works have been published [75,76], they rely on arbitrary (user-provided) instructions and (sequential/spatial) restrictions; in addition, only structures with previously known similar (experimentally solved) templates can be modeled. Moreover, the work toward an ab initio nucleic-acid-structure-modeling tool is complicated by the fact that UNa formation is often driven by additional (but important) factors like negative/positive supercoiling (in the case of DNA) [77], chromatin epigenetic marks [78], chemical modifications of nucleobases [79], molecular crowding conditions/local microenvironment [80,81], interacting proteins [82], and other (de)stabilizers. In other words, predicting nucleic acid structure can be paradoxically even more challenging than predicting the structures of proteins.

Experimental or modeled structures of UNas can be further inspected using virtual screening/high-throughput molecular docking to determine which known chemical or natural substances are capable of specific binding, as successfully applied, e.g., in the case of bimolecular human telomeric G-quadruplexes [83]. The resulting UNas and their binders can be further characterized using molecular dynamics methods [84],constituting a complementary approach to biophysical methods of wet-lab characterization. Unfortunately, another limiting factor here is the lack of user-friendly software that would allow a wide range of scientists to carry out these analyses independently. Considering the computational complexity of these analyses, an ideal solution could be an integrative web server allowing scientists to analyze UNa .pdb structures using natural-language commands with the help of artificial intelligence [85].

So far, for the entirety of the UNa entity, only G-quadruplexes have been considered the primary target in two clinical trials with CX-3543 and CX-5461 compounds [86,87]. The main pitfall of UNa-binding compounds has been low specificity and relatively high toxicity in vivo, as, for example, in the case of the known in vitro G-quadruplex stabilizer TMPyP4 [88]. Later, more specific compounds were developed, recognizing, e.g., only parallel or antiparallel types of G-quadruplexes [89]. We believe that advances in bioinformatic methods will soon allow the implementation of the well-known concept of one drug–one target in the field of UNas; this would allow selective targeting of particular pathological UNas that arise, for example, due to nucleotide repeat expansion [90,91]. Altogether, UNas represent very promising molecular targets, and the current boom in methods of computational biology can pave the way for their future application in drug discovery.

## Figures and Tables

**Table 1:** The selection of the most-researched UNas together with their basic characteristics, formation sequences, and known molecular-biological functions. Schematic structures were visualized using UCSF Chimera [16] based on experimentally solved structures in the case of A-DNA (PDB: 2RMQ), Z-DNA (PDB: 4OCB), and triplexes (PDB: 149D). For the remaining and more structurally complicated UNas, simplified diagrams were made using BioRender.

UNas	Schematic Structure	Basic Characteristics and Typical UNas-Forming Sequences	Molecular-Biological Function
A-DNA (A-RNA)	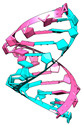	Short and wide helix Rigid, low flexibility No typical formation sequence Can arise under dehydrating conditions (e.g., desiccation of bacteria)	Most of double-stranded RNA is believed to exist in the A-form [17]. In addition, simple prokaryotic organisms and viruses can utilize this conformation to withstand adverse conditions [2,3].
Z-DNA (Z-RNA)	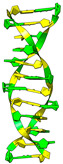	Left-handed nucleic acid Zig-zag phosphodiester backbone Alternation of purine and pyrimidine, typically d(CG)n or d(TG)n In vitro, it usually requires a high concentration of salts to be formed from B-DNA (4M NaCl)	Z-DNA and Z-RNA are associated with several human diseases, including various cancers and the autoimmune disease Aicardi–Goutières syndrome [18]. These UNas are also believed to play crucial roles in innate immunity and host–virus interactions [19]. Proteins preferentially recognizing left-handed nucleic acids are known [20,21,22,23].
Triplexes	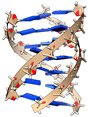	Triple-helical structure Can arise in sites of long homopurine and homopyridine tracts Prefers acidic or neutral pH and divalent cations	Sites of triplex-forming sequences are connected with microsatellite repeat expansion disorders, including Friedreich’s ataxia [24]. Although the in vivo formation of DNA triplexes has yet to be directly proved, it is believed to drive genomic instability [25].
R-loops	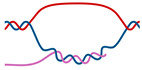	Three-stranded structure Consists of a DNA–RNA hybrid and a displaced strand of DNA Arises mainly in guanine-rich clusters	R-loops are formed during transcription when the nascent RNA strand base pairs with template DNA. They may cause genomic instability via the blocking of DNA replication [26]. R-loops are involved in the repair of double-stranded DNA breaks [27] and related to several human diseases [28].
G-quadruplexes	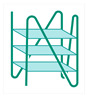	Guanines form planar G-quartets; these stack on each other Stabilized by monovalent cations (mostly K^+^ and Na^+^) Guanine-rich sequences (tracts) are usually separated by short loop-forming sequences	G-quadruplexes play vital roles in the replication [29], transcription [30], translation [31], telomere maintenance [32], and biogenesis of noncoding RNAs [33]. They are connected to many human diseases [34] as well as important physiological developmental processes [35].
i-motifs	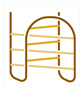	Formed by intercalated cytosine base pairs in slightly acidic conditions Cytosine-rich sequences, analogous to G-quadruplexes	i-motifs likely participate in transcription regulation [36]. In DNA, they can can arise in the opposite strand to the G-quadruplex [13]. There is also a hypothesis that i-motifs played an important role in the primordial RNA world [37].
Cruciforms	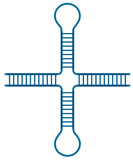	Structures consisting of a stem, branch point, and loop in the shape of a cruciform Can arise from inverted repeat sequences Stabilized by negative supercoiling	Cruciforms may play an important role in various biological processes, including replication [38], gene expression [39], recombination [38], and repair [40]. Inverted repeats that form cruciforms or hairpins are essential features of viral genomes, potentially driving their mutability [41,42].
